# Thermomechanical Properties of Nontoxic Plasticizers for Polyvinyl Chloride Predicted from Molecular Dynamics Simulations

**DOI:** 10.1021/acsami.3c02354

**Published:** 2023-05-11

**Authors:** Snigdha S. Jagarlapudi, Heaven S. Cross, Tridip Das, William A. Goddard

**Affiliations:** Materials and Process Simulation Center (MSC), California Institute of Technology, Pasadena, California 91125, United States

**Keywords:** Young’s modulus, shear modulus, diffusion constant, glass temperature, free volume

## Abstract

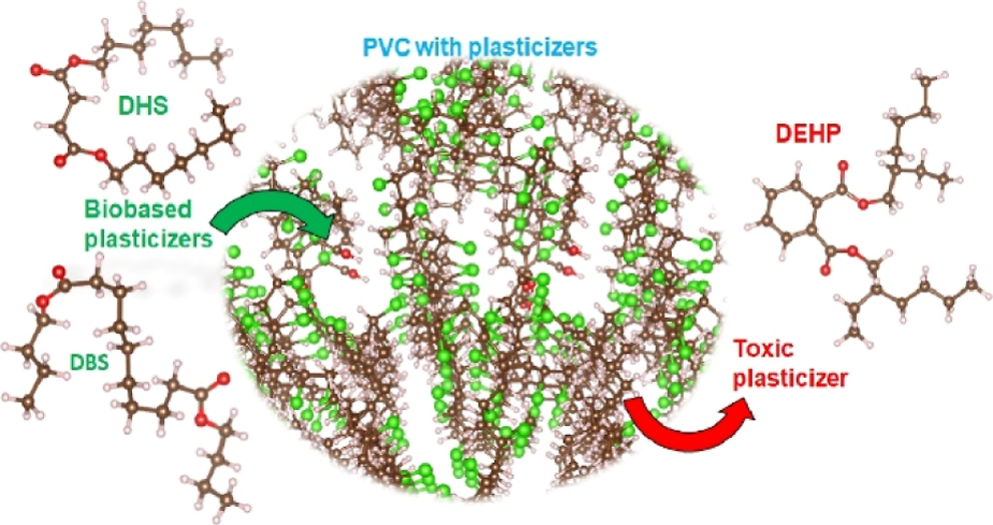

Environmental and toxicity concerns dictate replacement of di(2-ethylhexyl) phthalate (DEHP) plasticizer used to impart flexibility and thermal stability to polyvinyl chloride (PVC). Potential alternatives to DEHP in PVC include diheptyl succinate (DHS), diethyl adipate (DEA), 1,4-butanediol dibenzoate (1,4-BDB), and dibutyl sebacate (DBS). To examine whether that these bio-based plasticizers can compete with DEHP, we need to compare their tensile, mechanical, and diffusional properties. This work focuses on predicting the effect these plasticizers have on *T*_g_, Young’s modulus, shear modulus, fractional free volume, and diffusion for PVC–plasticizer systems. Where data was available, the results from this study are in good agreement with the experiment; we conclude that DBS and DHS are most promising green plasticizers for PVC, since they have properties comparable to DEHP but not the environmental and toxicity concerns.

## 1 Introduction

Polyvinyl chloride (PVC) plays a key role in the production of a large variety of products including building materials, furniture, toys, medical devices, electrical insulation, hygiene products, and food wrappers.^1^ However, pure PVC is naturally brittle causing issues during processing. To combat this issue, plasticizers are commonly added to the resin to impart flexibility and thermal stability.^2,3^

Traditionally, phthalates are the most widely used PVC plasticizer.^1^ In particular, PVC tubes using di(2-ethylhexyl) phthalate (DEHP) are used for external processing of blood and other bio-substances, presenting the concern that DEHP might leach into the blood to cause toxicity. Moreover, phthalates need to be eliminated because of serious environmental and public health concerns. In particular, DEHP has been found to induce a wide range of developmental and reproductive toxicities in mammals, and it is a suspected endocrine disruptor in humans.^4−7^ Directive 2005/84/EC of the European Parliament and Council addressed these alarming findings by restricting the use of DEHP from all toys and childcare products in the EU.^8^ Despite growing awareness of the harm that phthalates present, DEHP continues to be the most frequently used plasticizer in the world and can be found in commercial PVC in concentrations up to 30–40 wt %.^9^ In order to reduce the usage of DEHP and similar phthalate plasticizers, recent studies have focused on developing bio-based plasticizers without adverse effects that do not compromise the properties of the final product.^10,11^

In order to accelerate this development of new plasticizers, we use in silico techniques to predict the effect of new candidate plasticizers on the tensile and migration properties of PVC. We consider here four potential new plasticizers and compare their properties to DEHP. This in silico approach, in principle, could also be applied to a wide range of new candidates much faster than experimental synthesis and characterization, which would allow experiments to focus on the best predicted plasticizers.

In this study, we use a general computational approach that could be applied to a wide range of plasticizers, however, we focus on systems for which there is already some experimental data available. Indeed, we found that our predictions agree well with the available experiments, setting the stage for a broader sampling of possible plasticizers that have not yet been studied experimentally. Our studies indicate that dibutyl sebacate (DBS) and diheptyl succinate (DHS) plasticizers have properties comparable to DEHP but none of the toxic attributes of DEHP. Thus, we recommend the gradual switch from DEHP to DBS or DHS.

### 1.1 Effect of Structure on Plasticization

Plasticizers for PVC are used to enhance the flexibility and processability, which is achieved by lowering the glass transition temperature (*T*_g_).^2^

Many theories attempt to explain the mechanisms of plasticization, including gel theory, lubricity theory, and free volume theory.^12,13^ The free volume theory, proposed by Fox and Flory in the 1950s, is the most popular.^14^ At temperatures above the glass transition, the free volume can decrease continuously as the temperature is lowered, but below the glass transition temperature, diffusion within the polymer becomes increasingly slow causing excess volume. Adding a plasticizer, increases the free volume available by enabling increased motions in the polymer chains thus lowering the *T*_g_. Movements of polymer chain ends, chain sides, and main chain components increase as the temperature increases.^11,14^ The most effective plasticizers achieve this effect by having a relatively large branched molecular structure but a low molecular weight.^15^

**Figure 1:**
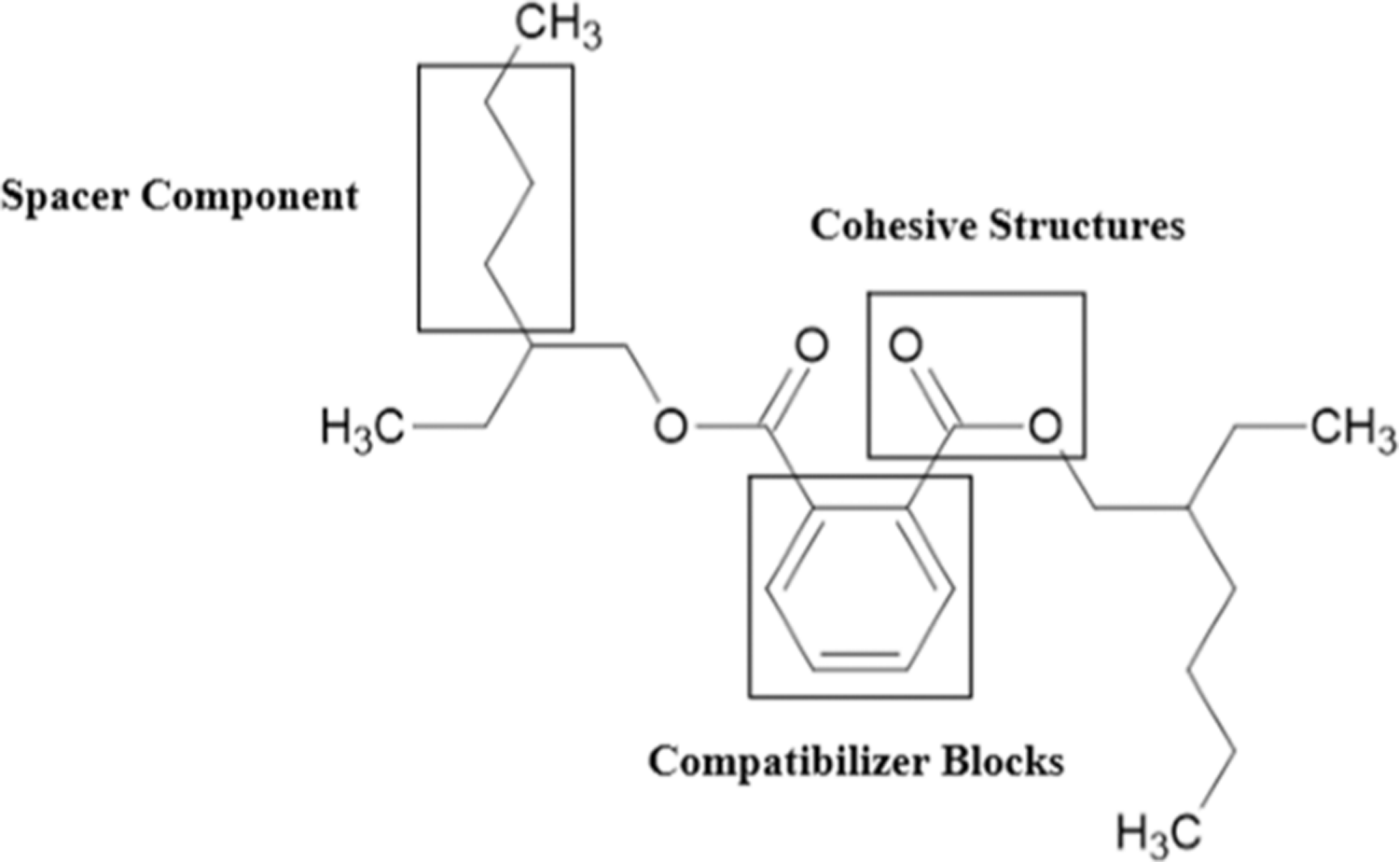
Structure of DEHP labeled to indicate spacer components, cohesive structures, and compatibilizer blocks.

The properties of plasticized PVC depend strongly on the interactions between the plasticizer molecule and the PVC backbone. Thus, polar groups cause polymer chains to be mutually attractive, increasing *T*_g_, while large nonpolar side chains tend to keep them apart, lowering *T*_g_.^14^ These interactions are classified into the three main components of a plasticizer: spacers, cohesive blocks, and compatibilizer blocks (Figure 1).^12^

Spacers are mainly aliphatic, dangling chains that favor nonpolar intermolecular interactions, adding extra free volume to the system to promote dynamic movement in the polymer chains. The most commonly used plasticizers contain an aromatic ring in their structures, which acts as a compatibilizer block.^12,16^ The presence of an aromatic ring introduces great flexibility to plastics. Ester groups within plasticizers act as cohesive structures due to van der Waals forces, hydrogen bonds, and electrostatic interactions that dominate their interactions with the PVC backbone. Critically, these bonds serve to prevent leaching and migration.^12,16−18^ This property is of great interest for developing eco-friendly and nontoxic alternatives to phthalates because the plasticizer molecule is not chemically bonded to a polymer chain. This makes them particularly susceptible to being released during the production process or during quotidian use.^12,15^

### 1.2 Previous Investigations into Bio-Based Plasticizers

A series of experiments investigated the *T*_g_ and tensile properties of PVC plasticized by fumarates, maleates, succinates, and adipates to determine their potential as bio-based plasticizers. They found that succinate and maleate plasticizers were able to lower *T*_g_ more effectively than DEHP. Furthermore, they concluded that only succinate plasticizers were able to lower Young’s moduli more effectively than DEHP at concentrations higher than 30 wt % while providing a higher rate of biodegradation.^19−22^ Stuart et al. examined succinate-based plasticizers further, finding that molecules with longer alkoxy chains, such as DHS, were the most effective at reducing Young’s modulus.^23^ These longer chains act as the spacer component of the plasticizer, introducing more movement and free volume into the plasticized system.^24^ The presence of the ester groups may make it less prone to migration.

Shirai et al. examined the plasticizing capabilities of adipate esters experimentally but in polylactic acid (PLA). They showed that diethyl adipate (DEA), which has a linear structure and a relatively small molecular weight, had the best plasticization effects on PLA.^25^ The cohesive structures present in DEA indicate that it may also be resistant to diffusion; this combined with positive results for plasticization in PLA, makes DEA a viable candidate for use in PVC.

The diol dibenzoate family of compounds is of interest in the search for viable alternative plasticizers because diethylene glycol dibenzoate (DEGDB) is already in wide commercial use. However, there is concern that its ether linkages may lead to the formation of metabolites that are difficult to degrade microbially. These metabolites, similar to the formation of MEHP in DEHP, could be more toxic than its parent compound. 1,4-Butanediol dibenzoate (1,4-BDB) is an interesting alternative to DEGDB because it eliminates the ether functional group that poses a risk.^26^ 1,4-BDB was also shown to be biodegradable by soil microorganisms.^26−28^ Furthermore, an in vivo rat study showed that 1,4-BDB does not significantly alter adult male reproductive function.^29^ The 1,4-BDB structure has two aromatic rings which can act as compatibilizer bocks to impart flexibility and two ester groups that can act as cohesive blocks to prevent leaching.

Another class of chemicals of interest is the sebacates. While the tensile properties of sebacate plasticizers in PVC have not been studied, Mahnaj et al.^30^ studied their effectiveness in ethyl cellulose polymer. They found that dibutyl sebacate was the most successful at lowering T_g_ among the sebacate-based plasticizers studied. DBS also has the longest aliphatic chains compared to the aforementioned plasticizers, suggesting that these spacer components will generate more free volume and increase plasticization.

The above results show the promise of diheptyl succinate, diethyl adipate, 1,4-butanediol dibenzoate, and dibutyl sebacate as potential alternatives to DEHP in PVC (Table 1). In addition, numerous investigations have been made into other potential bio-based plasticizers. One promising class of candidate is the cardanol-derived plasticizers, such CHE-12 and cardanol acetate. Both have been shown to be nontoxic while having higher plasticizer compatibility with PVC compared to the toxic commercial plasticizers DOP and DINP. Although our current study does consider such plasticizers, future studies should investigate their compatibility and efficiency with PVC.^31−33^

**Table 1:**
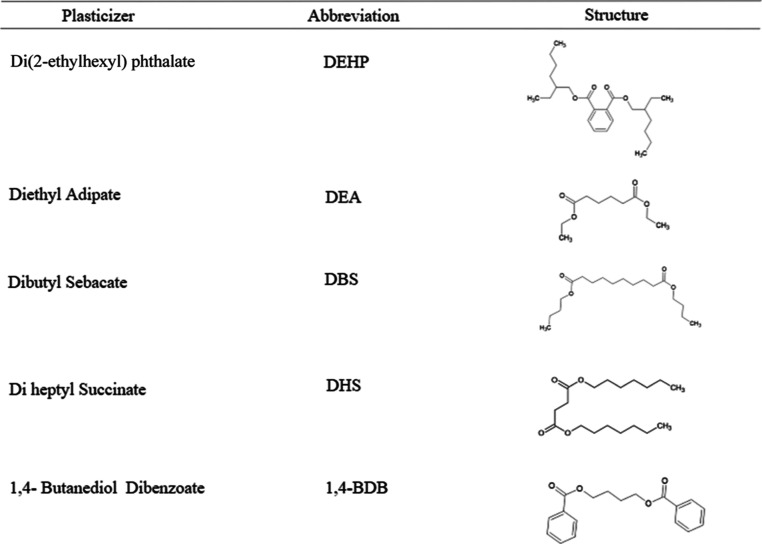
Structures of the Plasticizers Studied

In order to ensure our chosen bio-based plasticizers can compete with DEHP, we need to examine their tensile, mechanical, and migration properties. This work focuses on predicting the effect these plasticizers have on *T*_g_, Young’s modulus, shear modulus, fractional free volume, and diffusion for PVC systems.

These blended polymer properties can be determined experimentally, however, such experimental tests are resource and time expensive and most have not yet been reported for our 4 candidates. Instead, we use classical molecular dynamics (MD) simulations as a practical approach to predict such properties, enabling in silico predictions to identify the most promising candidates for future experimental validation. MD also allows investigation into the mechanisms connecting atomistic structure with diffusion and tensile properties. These properties depend heavily on a diverse set of factors including free volume, density, pressure, temperature, and the interaction of the plasticizer with the PVC backbone, all of which can be monitored systematically throughout the simulation. Thus, our use of MD is two-pronged: to maximize efficiency and to exact more control and monitoring within the simulation. Our MD simulations predict the dynamics of atomic movements based on force fields fitted to quantum mechanics (QM). This enables practical calculations of the atomistic behavior and various-mechanical properties of novel polymer blends. In order to validate these in silico methods, we compared our predicted results to experimental findings where available. Indeed our results are within the same order of magnitude as what was reported, indicating a good agreement. It would be valuable to obtain experimental data for the mechanical and transport properties for these novel PVC-plasticizer blends.

## 2 Simulation Methods

All MD calculations were carried out using the LAMMPS software.^34^ We used the universal force field (UFF), which includes parameters for every atom of the periodic table (up to atomic number 103).^35^ This allows for MD calculations of organic, biological, and inorganic molecules and solids. UFF describes the potential energy of a system as in eq 1
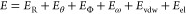
1where the valence interactions include bond stretching (*E*_R_), bond bending (*E*_θ_), dihedral torsion (*E*_Φ_), and inversion terms (*E*_ω_). The nonbonded interactions consists of van der Waals (*E*_vdw_) and electrostatic interactions (*E*_el_).

We used an MD time step of 1 fs. All simulations were conducted with a temperature damping constant of 100 fs while the NPT MD used an isotropic barometer with a pressure damping constant of 1000 fs.

### 2.1 Model Construction

We built the molecular structures using Materials Studio with the OPLS2005 forcefield parameters.^36^ We first built a system of pure PVC using the “Amorphous Cell Builder” in Maestro. Considering commercial PVC varies greatly but generally has an atactic stereochemistry, we chose a system consisting of 16 atactic-PVC chains with 20 monomers each. This system, along with the plasticizer of choice, was loaded into the “Disordered System Builder”. All systems were carried out with a plasticizer composition of ∼40 wt % as this is the rough upper limit for the amount of DEHP used in commercial PVC. Exaggerating the amount of plasticizer present in the system allows for more obvious observation of plasticization properties.^9^

We followed the MD equilibration procedure outlined by Li et al.^37^ to ensure full interspersion of the plasticizer molecules within the system. This protocol included the following steps:

- Energy minimization at a low density (0.5 g/cc) followed by an 8 ns MD with the volume fixed but with the temperature controlled by a thermostat (denoted as *NVT*) to heat the system to 600 K and equilibrate.
- Next, we gradually increased the density of the system to 0.8 g/cm^3^ over a period of 1 ns.
- Once the target density was reached, the temperature was reduced to 300 K and equilibrated for 3 ns with the temperature controlled by a thermostat and the pressure controlled by a barostat (denoted as *NPT*) at 1 atm.
- The final step was 5 repeated heating–cooling cycles each with an 8 ns *NPT* MD at 600 K followed by a 5 ns *NPT* MD at 300 K, both at 1 atm. This was continued until the final density converged.

After convergence, the pressure fluctuated around zero indicting equilibration. The time evolution of the potential energy, pressure, and temperature are found in Figures S1–3 in the Supporting Information (SI), respectively. The predicted densities are compared with experiment and shown in Table 2.

**Table 2:** Predicted Density of PVC/Plasticizer Blends

					density		
plasticizers	molecular weight (g/mol)	percent weight (%)	mole ratio (plasticizer/PVC)	number of atoms in system	calculated	experimental	% error
pure PVC	62.5^a^			1984	1.338 ± 0.051	1.345^38^	2.763 ± 2.637
di(2-ethylhexyl) phthalate (DEHP)	390.55	40.3	0.108	3521	1.242 ± 0.023	1.216^39^	2.129 ± 1.555
diheptyl succinate (DHS)	314.76	39.8	0.131	3440	1.294 ± 0.034	no data	
dibutyl sebacate (DBS)	314.46	40.9	0.137	3904	1.356 ± 0.042	no data	
diethyl adipate (DEA)	174.19	39.6	0.130	4160	1.347 ± 0.039	no data	
1,4-butinedal dibenzoate (1,4-BDB)	298.33	40.4	0.134	3904	1.301 ± 0.058	no data	

a Refers to one repeating unit of PVC.

### 2.2 Young’s Modulus

The Young’s modulus or elastic modulus, *E*, is the slope of the initial linear part of the stress–strain curve.^37^ As the amounts of plasticizer are increased, the polymer system has greater chain mobility, leading to less resistance to deformation. This results in a Young’s modulus that decreases with increasing plasticizer concentration, which is desired in commercial plastics. The Young’s modulus also generally decreases with increasing temperature.^40^

We examined the Young’s modulus as a function of temperature, *E*(*T*). To do this, we calculated elastic moduli by deforming each system in the PVC chain direction (*z*) at a constant engineering strain rate of 0.0001 ns^–1^ up to a total strain of 1% for various temperatures over the range of 150–500 K. We heated (or cooled) the system to the desired temperature using *NVT* MD. The system was then equilibrated at the new temperature at 0 atm pressure for 0.5 nanoseconds using *NPT*. The final step before deformation was a brief relaxation period using the *NPT* ensemble, where the *x* and *y* directions were allowed to relax completely using anisotropic barometer conditions.

The six stress components (Σ*xx*, Σ*yy*, Σ*zz*, Σ*xy*, Σ*yz*, Σ*xz*) were fitted to the Von Mises Criterion (eq 2) to account for the monocrystalline nature of the simulation boxes. The final stress as a function of the applied strain leads to a slope that is equivalent to Young’s modulus.^41−43^
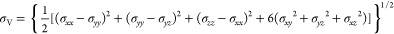
2

### 2.3 Shear Modulus

The shear modulus, *G*, provides information about the rigidity of a material. It is defined as the ratio of shear stress to the shear strain. This is of interest in commercial plastics because it indicates the material’s resistance to shearing and torsional forces.^44^ We calculated the shear modulus as a function of temperature, *G*(*T*).

We used a protocol similar to that for *E*(*T*). Since the PVC chains are oriented in the *z* direction, we sheared each system in the *yz* and *xz* planes at a constant engineering shear strain rate of 0.0001 ns^–1^ from 150 to 500 K.

As with *E*(*T*), we heated (or cooled) the system to the desired temperature using the *NVT* and then equilibrated at the new temperature for 0.5 nanoseconds at 0 atm pressure using *NPT*. Prior to deformation we equilibrated using *NPT*, allowing the *xx*, *yy*, *zz*, and *xy* planes to relax completely.

The *xz* and *yz* planes were sheared independently. The Von Mises Criterion equation was again fitted to the pressure components. The resulting shear stress value was evaluated as a function of the applied shear strain. Then, the two stress values calculated from shearing in the *xz* and *yz* planes were averaged and plotted against the shear strain, to find the overall shear modulus of the system.

### 2.4 Fractional Free Volume

In order to validate the glass transition temperatures obtained in Sections 2.2 and 2.3, we calculated the fractional free volume (FFV) which characterizes free volume in polymers. It can be calculated with the following empirical equation^45^
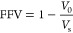
3where *V*_s_ is the specific volume of the polymer and *V*_0_ is the occupied volume, which is equivalent to 1.3*V*_w_. *V*_w_ is the van der Wall’s surface of the polymer, which can be calculated by^46^
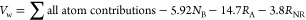
4where *N*_B_ is the number of bonds, *R*_A_ is the number of aromatic rings, and *R*_NR_ is the number of nonaromatic rings. FFV for the polymer blends were calculated for temperatures between 250 and 425 in 25 intervals. These temperatures cover the broad range of glass transition temperatures expected for PVC blends.

### 2.5 Diffusion

Plasticizers migration is critically important to potential commercial uses of the PVC polymer and to public health. We calculated the mean square displacement (MSD) as a function of time to get the diffusion coefficient, *D*, from the Fickian relation
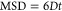
5

The MD procedure to obtain MSD is as follows:^47^

- To generate initial velocities for the atoms in simulations, we carried out our *NVT* simulations at 20 K for 10 ps.
- The system was then heated from 20 K to room temperature (300 k) over 0.5 ns using *NVT*. MD simulations were also carried out at 400, 500, 600, 700, and 800 k, each for ∼15–20 ns, allowing us to predict the activation energy for diffusion of the additive. This range of temperatures was chosen to allow sufficiently long simulation times at the higher temperatures to attain the Fickian diffusion limit (eq 5).
- Then we carried out *NPT* simulations at the target temperature for 1 ns to release any residual stress from heating the structure.
- Finally, *NVT* simulations were carried out for ∼15–20 ns at each temperatures, while applying the Berendsen thermostat (damping time = 0.1 ps).
- The pressure and density fluctuations during the simulations are reported in Figures S1–S18 of the Supporting Information.
- Spatial coordinates and time step data for all the atoms were saved every ps from the 15 to 20 ns trajectories.
- A python code was used to calculate the MSD vs time step (τ) as in eq 6. That is for each time increment, we average the MSD(*t*) over all *t* increments along the trajectory:
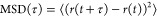
6

The diffusion coefficients as a function of *T* were used to obtain the activation barrier, *E*_a_ (eq 7). The Log MSD vs Log *t* plot must be tangent to the linear line to obtain *D*. This was true for higher temperatures, which were extrapolated to room temperature to obtain *D* at 298 K. Extrapolation was used to predict the diffusion at 298 K considering the simulation time to attain the Fickian limit, eq 5, would be too long to be considered feasible.
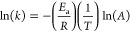
7

## 3 Results and Discussion

### 3.1 Young’s Modulus

**Figure 2:**
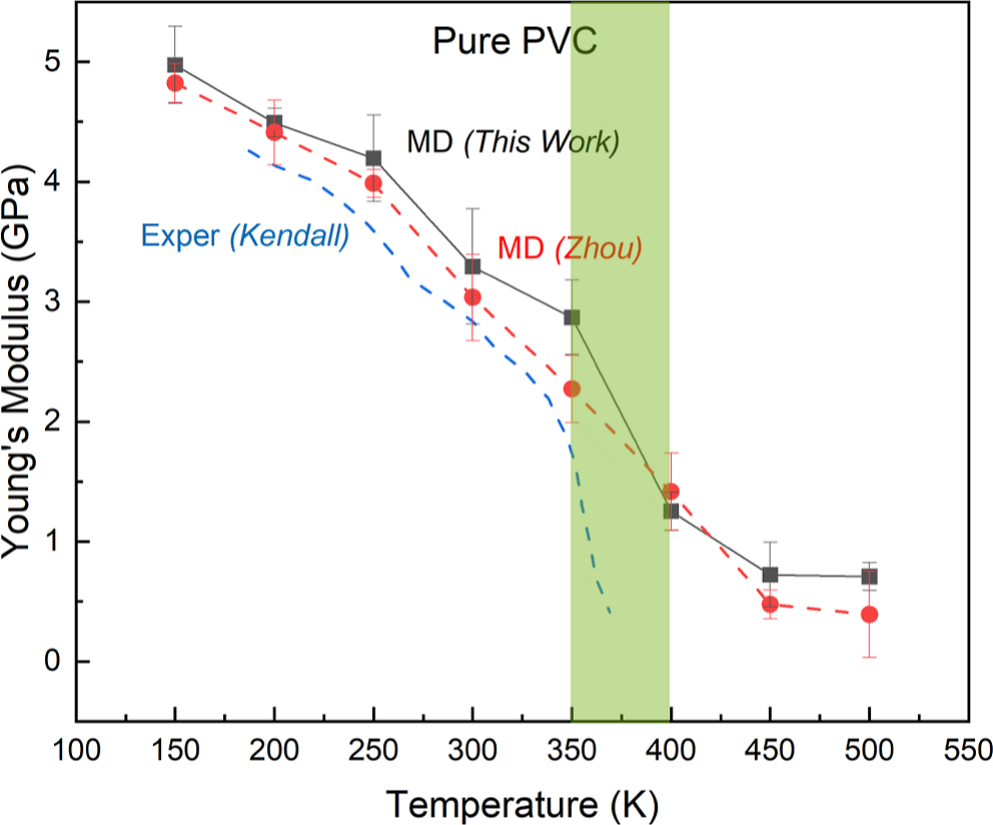
Predicted Young’s modulus for pure PVC at temperatures from 150 to 500 K at a strain rate of 0.0001 ns^–1^. The green shaded box highlights the significant drop between 350 and 400 K. Our predicted *E*(*T*) is compared to experiment from Kendall and Siviour^48^ at a strain rate at 0.01–1 s^–1^ and to MD results by Zhou and Milner^49^ at a strain rate of 0.04 ns^–1^ with a maximum strain of 4%.

In order to ensure that our calculational protocol leads to an accurate *E*(*T*), we compare in Figure 2 our simulation results to experimental data and to other molecular dynamics calculations. Our predicted elastic moduli for pure PVC and 40 wt % DEHP are in reasonable agreement with existing results.

We found no literature regarding PVC blends with DBS, DEA, and 1,4-BDB.

**Figure 3:**
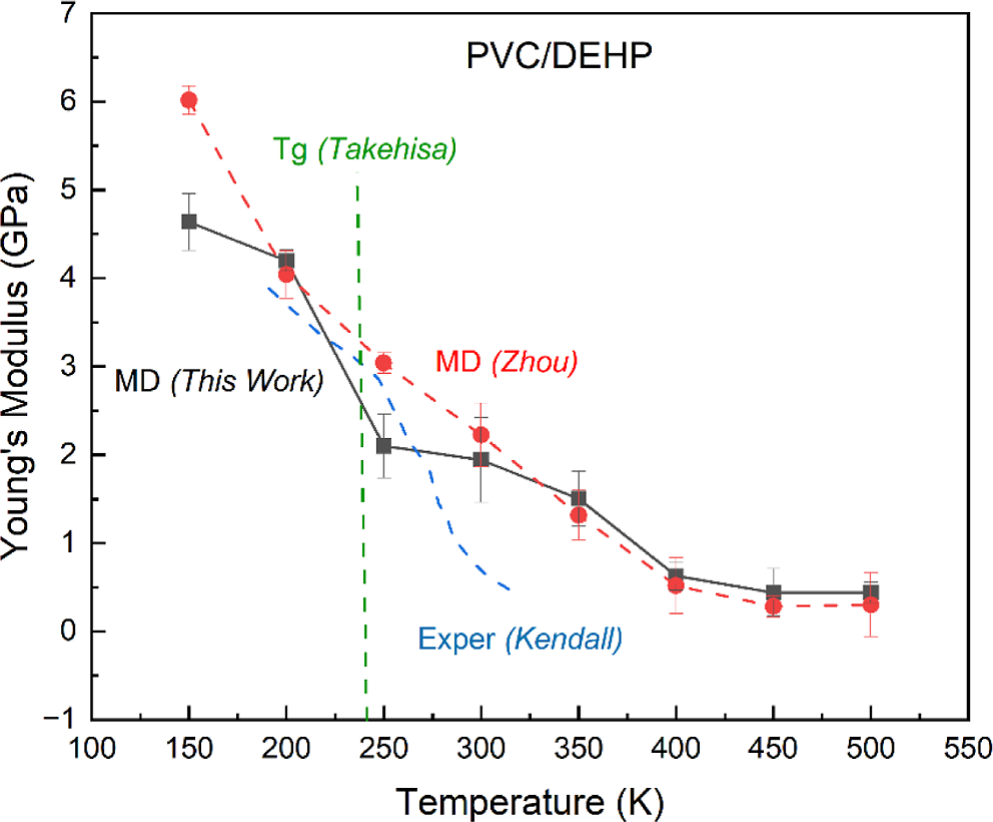
Predicted Young’s modulus *E*(*T*) at a strain rate of 0.0001 ns^–1^ for PVC plasticized with 40% DEHP at temperatures from 150 to 500 K. This shows a big decrease between 200 and 250 K, in good agreement with experimental glass transition temperature found by Takehisa et al. of a PVC/DEHP system with a similar weight percent.^51^ Also compared are the experiments by Kendall and Siviour^48^ at a strain rate of 0.01 to 1 s^–1^ for a PVC system plasticized with 40 wt % DINP and MD results by Zhou and Milner^49^ at a strain rate of 0.04 ns^–1^ with a system plasticized with 39.4 wt % DEHP.

Our calculated *E*(*T*) for 40 wt % DEHP is higher than the values measured by Kendall and Siviour^48^ which have 40 wt % DINP and those calculated by Zhou and Milner^49^ which have 40 wt % DEHP (Figure 3). This positive deviation from expected behavior suggests that the plasticizers and polymer bind more strongly to each other compared to the average of the pure component interactions.^37^

**Figure 4:**
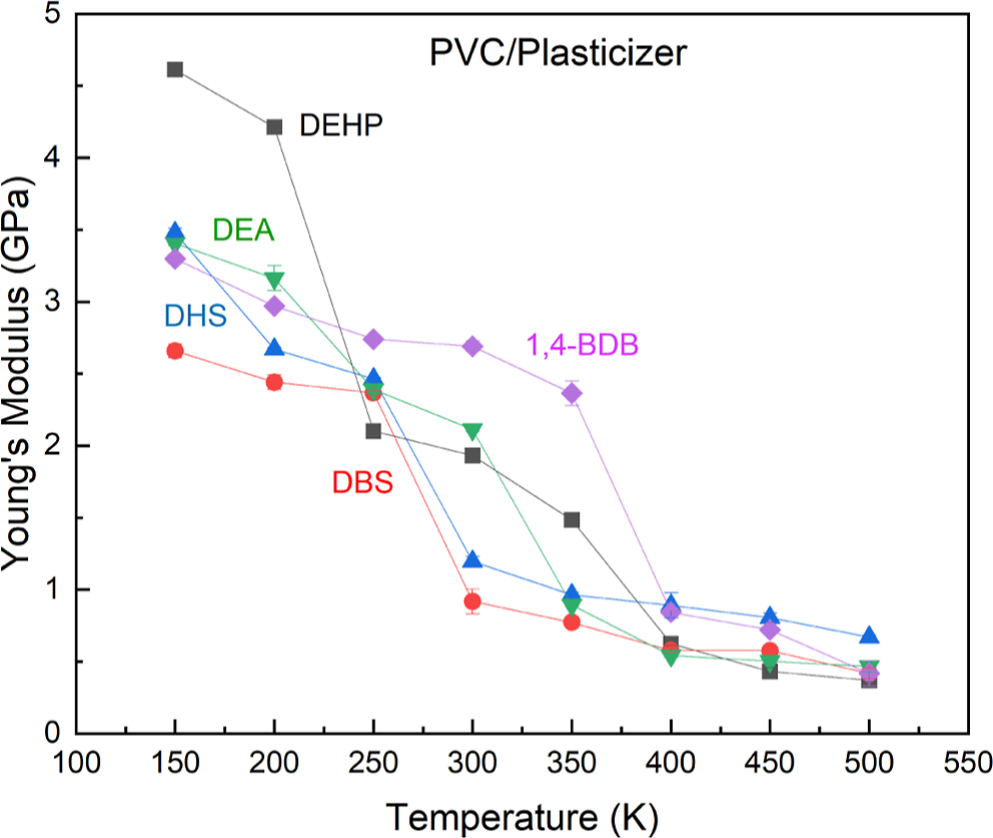
Predicted Young’s modulus for systems of PVC plasticized with DEHP, DBS, DHS, DEA, and 1,4-BDB at temperatures from 150 to 500 K. This indicates that the glass temperature for DBS and DSE is between 250 and 300 K and above 300 K for DEA, DEHP, and 1,4-BDB.

The *E*(*x*) of all plasticized systems converged as the temperature reached 500 K. DBS was the only plasticizer that consistently decreased the Young’s modulus of PVC as well as or better than DEHP (Figure 4).

We expected DBS to substantially increase the flexibility of PVC due to its two aromatic rings whose polarity increases the interaction with the PVC backbone. Indeed DBS and DHS were the only plasticizers that lead to a lower Young’s modulus than DEHP at room temperature (∼298 K).^15^

A dramatic decrease in *E*(*x*) at some temperatures was observed in all systems. We used this change in slope as an indicator of glass transition temperature, since the glass transition region is the point at which increased movement of carbon chains is observed, causing the polymer to shift from a rigid to rubbery state. Since the purpose of a plasticizer is to increase the chain mobility, *T*_g_ has been demonstrated to be a good indicator of the polymer structure and efficacy. We showed that DEHP lowers the *T*_g_ of PVC by almost 100 K, from between 350 and 400 K to between 250 and 300 K. DBS and DHS were the only other plasticizers able to lower the *T*_g_ to such an extent. DEA, despite its lowmolecular weight, was able to reduce the *T*_g_ by only 50 K to between 300 and 350 K. Elsiwi et al. determined that DHS has a *T*_g_ of 248.15 K at a 26.8 wt % This substantially lower percent weight may explain the deviation of our predicted results from experiment.^24^

The evident outlier among our results is 1,4-BDB that we found to not decrease either the *T*_g_ of PVC or the *E*(*x*) at temperatures above 250 K. This apparent lack of plasticization was also observed in an experimental study conducted by Erythropel et al*.*^26^ One proposed hypothesis to explain this anomaly is that the stacking of the two benzyl groups along with 1,4-BDB spacer components caused rapid partial crystallization within the blend, therefore losing its ability to plasticize PVC.

### 3.2 Shear Modulus

**Figure 5:**
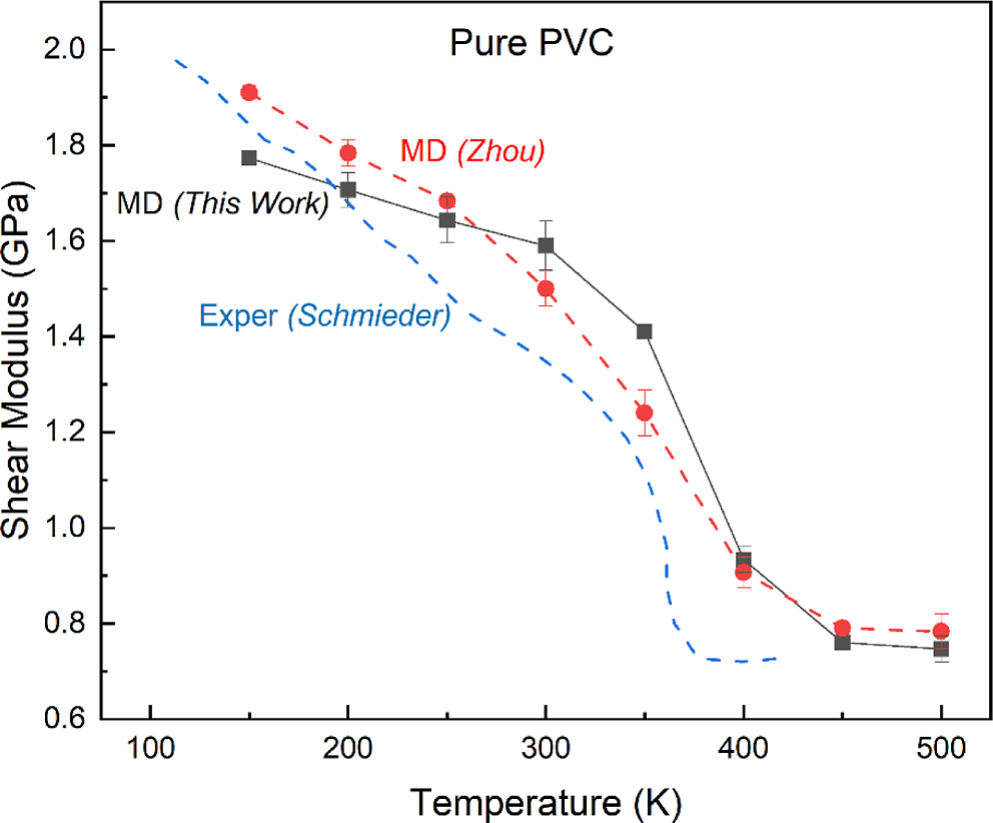
Predicted shear modulus for pure PVC at temperatures from 150 to 500 K at a strain rate of 0.0001 ns^–1^. This is compared with experiments by Schmieder and Wolf^52^ at a strain rate at 0.01–1 s^–1^ and MD simulations by Zhou and Milner^49^ at a strain rate of 0.04 ns^–1^ with a maximum strain of 4%.

In order to ensure that the protocol used in this work would accurately predict the shear modulus, *G*(*T*), we compare our MD results to experimental data for pure PVC in Figure 5. We could not find experimental shear modulus data in the literature for PVC blends with DEHP, DES, DBS, DEA, and 1,4-BDB. We found that the shear modulus calculated when shearing in the *xz* plane is similar to that when shearing in the *yz* plane for each system, so we quote the average value here.

**Figure 6:**
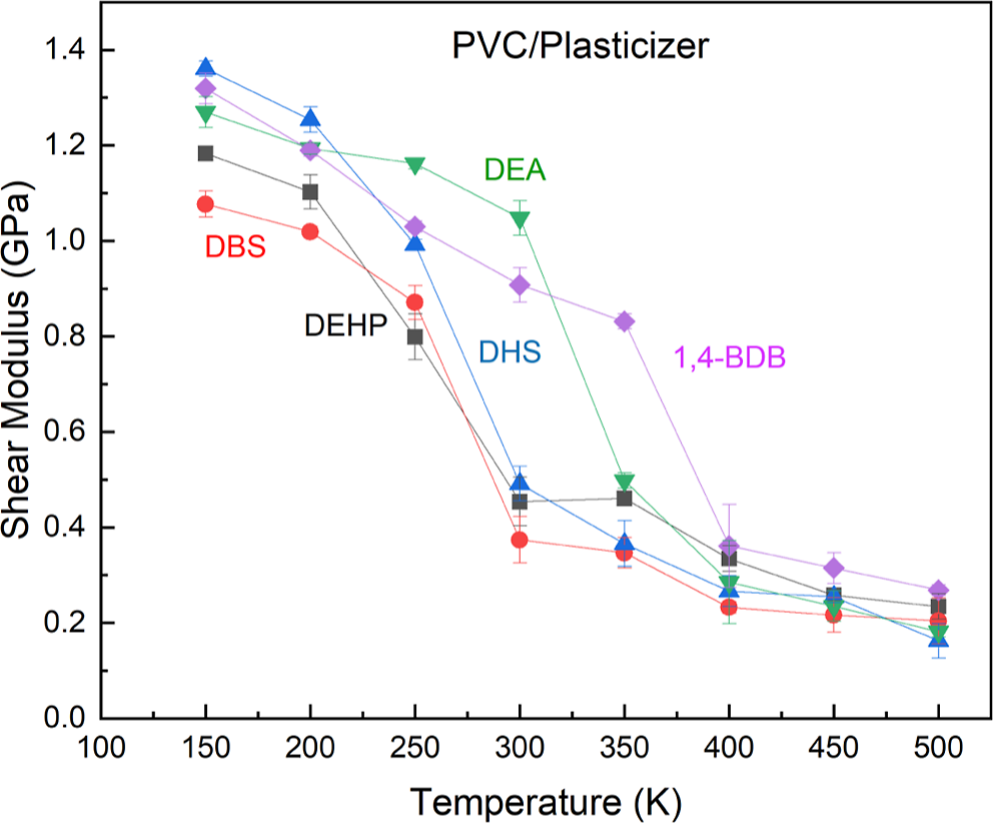
Predicted shear modulus for PVC plasticized with DEHP, DBS, DHS, DEA, and 1,4-BDB at temperatures from 150 to 500 K. The large change in the range 250 and 300 K for DBS, DEHP, and DSE can be associated with the glass temperature as can the large change from 300 to 350 K for DEA and the change from 350 to 400 K for 1,4-BDB.

The results obtained from shear simulations corroborate the findings for the Young’s modulus. The glass transition temperatures shown in the *G*(*T*) of the various plasticizer systems demonstrate that DEHP, DBS, and DHS all successfully lower the glass transition temperature of PVC to between 250–300 K (Figure 6). DBS was the only plasticizer to consistently lower the *G*(*T*) of PVC as well as or better than DEHP. 1-4-BDB continued to exhibit anomalous behavior that suggested an inability to plasticize PVC (at least at this concentration).

### 3.3 Fractional Free Volume

In order to validate the *T*_g_ predicted from the Young’s and shear modulus, we calculated the fractional free volume, FFV. FFV is a measure of the volume not occupied by the polymer, making it an indicator of plasticization efficiency.^45^ It has been shown that thermal expansion increased free volume and at a certain temperature, the mobility PVC backbone increased transforming it into rubber. This rubber transition boundary can be interpreted as the glass transition temperature.^53^ We calculated the FFV in the PVC–plasticizer systems as a function of temperature to estimate *T*_g_.

**Figure 7:**
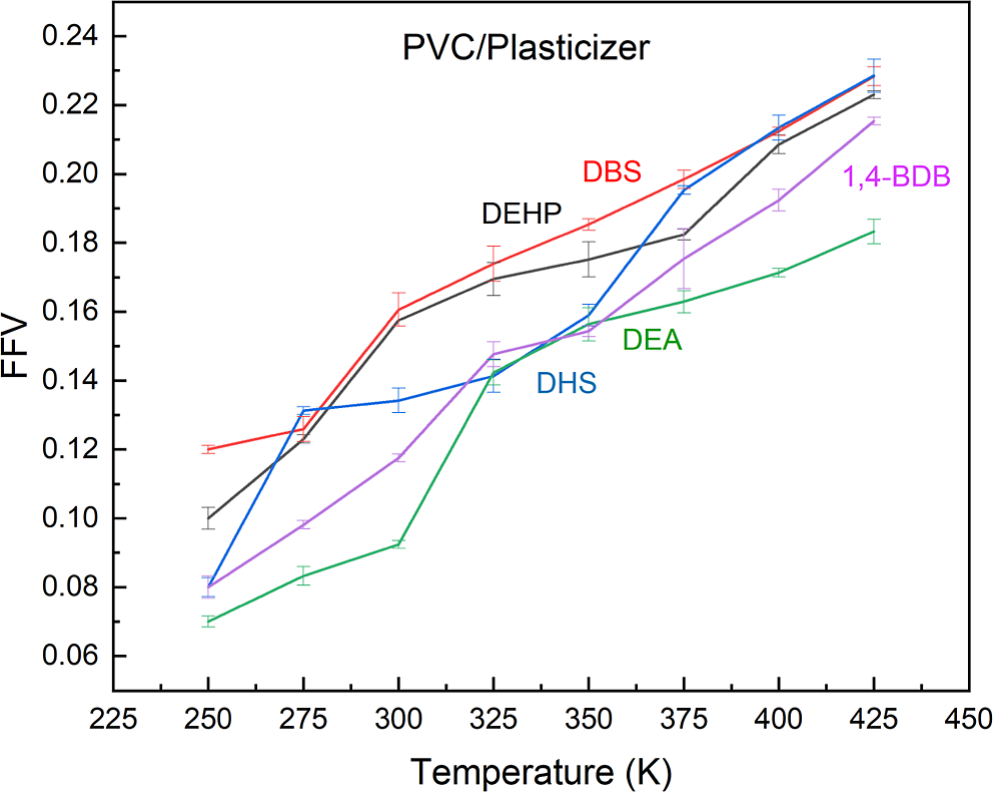
Calculated fractional free volume for PVC plasticized with DEHP, DBS, DHS, DEA, and 1,4-BDB at temperatures from 250 to 425 K.

We observed that the predicted FFV values correspond well with the previously predicted *T*_g_ values based on the shear behavior. A sudden change in slope was observed between 275 and 300 K for the PVC/DEHP and PVC/DBS blends while a similar phenomenon was observed between 250 and 275 K for DHS. DEA exhibited a glass transition temperature between 300 and 325 K and 1,4-BDB once again demonstrated the highest *T*_g_. However, Figure 7 shows that 1,4-BDB exhibits a sudden change in slope between 325 and 350 K, a lower value than found from tensile simulations. One possible explanation for this observed difference stems from the calculation of *V*_w_. This approach takes into account “dead volume” in the repeat units of the chain which can depend on the conformation so that its use to estimate *V*_0_ cannot reliably describe polymers at room temperatures.^54^

Nonetheless, the prediction based on FFV values support the previous conclusion that DBS and DHS blends perform at similar or even better levels than DEHP at increasing chain mobility and plasticization.

### 3.4 Diffusion

**Figure 8:**
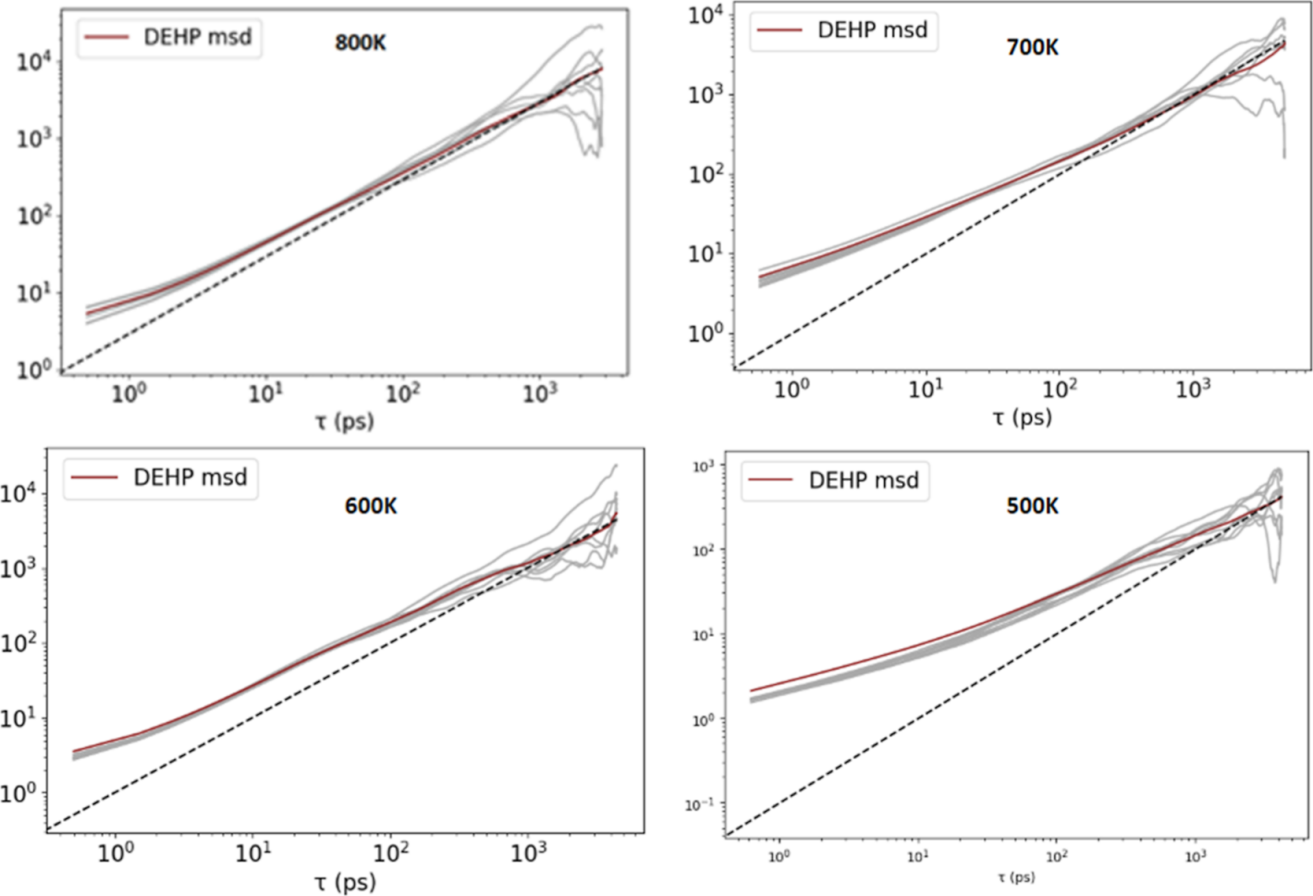
The log (MSD) vs log (time) plot to extract the diffusion constant for DEHP in PVC system at 500–800 K. The dashed line shows the Fickian relation, MSD = 6*D* × time, which should be tangent to the calculated log MSD vs log time curve. This system has 8 DEHP molecules, the diffusion of which are all tracked individually and then averaged to get the diffusion coefficients (red DEHP MSD line).

We established the validity of our diffusion results by ensuring that the MD was sufficiently long to reach the Fickian Diffusion regime, where log MSD is tangent to log(*t*) (and with the pressure fluctuating around zero). The MSD vs Time plots of all the systems investigated reached the Fickian diffusive regime, however the time required depended on the system and the temperature (Figure 8). The system pressure was consistently stable during the simulations. The activation energy, *E*_a_, was extracted from the slopes of the Arrhenius plots and used to extrapolate *D* to 300 K. We found that the diffusion coefficients for temperatures ranging from 300 to 800 K lead to two different ranges for the activation energies, *E*_a_ (Figures S18–S22). The Arrhenius plots for all five systems showed a change in slope between 600 and 700 K. This temperature range has been associated with the de-hydrochlorination of the PVC backbone, which may explain the increase in *D* observed in the plots.^55,56^ The MSD vs time, pressure vs time, and Arrhenius plots for each calculation are in Figures S4–S22 of the Supporting Information.

The diffusivity of the additives involves many variables, including interactions between the additive molecules and the polymer, the movement of the within the polymer, and the molecular size/structure of the plasticizer itself.^15^ We found that only DEA has a larger diffusion coefficient than DEHP at room temperature, while DBS, DHS, and 1,4-BDB all have high coefficients within a similar range (Table 3). DEA is an excellent solvent for PVC as it considerably increases plasticization properties and eases the production process, but DEA leads to high diffusion rates due to its polarity.^55^ Increased plasticizer polarity further leads to an increase in the probability of dehydrochlorination, which can lead to the degradation of the PVC system. As the system degrades, the migration of DEA molecules increases substantially.^55−58^ The tendency of plasticizer to remain in the plasticized material is also dependent on molecular size, the larger the plasticizer molecule the greater its permanence.^15,55^ This proclivity can be explained by the free-volume theory: larger pockets of free space are required for larger molecules. Moreover, there is an inverse relationship between molecular size and the diffusion rate.^58−60^ Thus, low diffusivity can be achieved by high molecular weight and highly branched isomeric structures, as exhibited by DBS, DHS, and 1,4-BDB. They contain ester groups, whose van der Waals forces, hydrogen bonds, and electrostatic interactions dominate the interactions with the PVC backbone, leading to lower diffusion rates for DBS, DHS, and 1,4-BDB.

**Table 3:** Predicted Diffusion Coefficients of Plasticizers at Temperatures Ranging from 300–800 K

	diffusion coefficients, *D* (m^2^/s)				
plasticizers	300 K^a^	500 K	600 K	700 K	800 K
di(2-ethylhexyl) phthalate (DEHP)	2.269 × 10^–14^	4.436 × 10^–12^	1.282 × 10^–11^	1.060 × 10^–^^9^	3.038 × 10^–^^9^
diheptyl succinate (DHS)	5.984 × 10^–15^	6.803 × 10^–11^	2.883 × 10^–10^	1.260 × 10^–^^8^	8.373 × 10^–^^8^
diethyl adipate (DEA)	3.985 × 10^–12^	1.873 × 10^–12^	3.812 × 10^–11^	2.652 × 10^–^^9^	4.721 × 10^–^^9^
dibutyl sebacate (DBS)	1.637 × 10^–15^	4.303 × 10^–12^	6.873 × 10^–12^	1.160 × 10^–^^9^	4.873 × 10^–^^9^
1,4-butanediol dibenzoate (1,4-BDB)	2.885 × 10^–15^	3.125 × 10^–10^	7.872 × 10^–10^	3.274 × 10^–^^7^	3.883 × 10^–^^7^

a Extrapolated using the Arrhenius equation.

The experimental data on diffusion are based on the diffusion of DEHP from a sheet of PVC into a mixture of water, ethanol, and/or acetonitrile, which cannot be compared directly to our predicted diffusion coefficient. In contrast, our study examines directly the diffusion of a plasticizer within the PVC. The Kim et al.^61^ experimental study found the diffusion coefficient of PVC plasticized with 40 wt % DEHP at 40 °C to be 4.77 × 10^–10^ or 5.75 × 10^–10^ m^2^/s depending on the solvent in which the PVC was submerged. While this value is within the same order of magnitude as our predicted value, the Kim study and the vast majority of experiments calculate the diffusion of DEHP from a sheet of PVC into a mixture of water, ethanol, and/or acetonitrile, which cannot be compared directly to our predicted diffusion coefficient.

## 4 Summary and Conclusions

After extensive literature search we considered four bio-based plasticizers as potential alternatives to using DEHP in PVC: DBS, DHS, DEA, and 1,4-BDB. For all four systems and for pure PVC and DEHP, we carried out MD simulations to predict their mechanical and diffusional properties. There are two advantages to using atomistic simulations:

- identify promising plasticizers without the excess resources and time required to carry out experiments and
- to identify the important atomistic factors affecting the properties of interest.

Our simulations used a PVC backbone consisting of 16 chains with 20 monomers each. However commercial PVC has a variety of compositions. Discrepancies found between our predicted results and experimental studies may arise from this difference in frameworks. Future in silico investigations could consider using single bi-populated chains of PVC; since studies have shown that this minimizes the impact of chain end attractions. Our studies used 40 wt % plasticizers, which is at the upper boundary of the amounts used commercially. This was chosen to exaggerate the effects of the plasticizers for more obvious comparisons. The amount of experimental data to validate our predicted values was limited, however where available we found good agreement. This work serves as a baseline for comprehensive atomistic investigations into plasticizers. However, future experimental studies would be useful to corroborate our findings.

In order to quantify the effect of the polymer blends on mechanical properties, we predicted Young’s modulus, shear modulus, fractional free volume, and diffusion coefficients. The consensus from this series of simulations on bulk moduli, diffusion, and thermal stability is that the plasticizer performance of DBS and DHS in PVC are both is at the same level or better than DEHP. Furthermore, both DBS and DHS have been shown to be nontoxic while DHS is biodegradable and can be sourced from renewable feedstock. This leads us to conclude that DHS and DBS are excellent candidates for replacing DEHP as a plasticizer. In practice, other knowledge of other properties is required to ensure suitability for industrial production. Using the aptitude dibutyl sebacate and diheptyl succinate demonstrated in this work, properties like viscosity, bulk moduli, thermal stability, and impact on color can be explored. In summary, this work sets the stage for future experimentation into increasing the sustainability of commercial PVC plastics.

## Data Availability

The data that support the findings of this study are available from the corresponding author upon request.

## Supporting Information Available

Supporting Information includes equilibration data for all systems and complete set of MSD, pressure vs time, and Arrhenius plots (PDF)
