# Supplementary material for: Thermomechanical Properties of Nontoxic Plasticizers for Polyvinyl Chloride Predicted from Molecular Dynamics Simulations
Source: ACS Appl Mater Interfaces. 2023 May 11;15(20):24858–67. doi: 10.1021/acsami.3c02354 (PMC10214373; doi:10.1021/acsami.3c02354)
Supplement: Supplementary file 1 — am3c02354_si_001.pdf [file am3c02354_si_001.pdf]

Supporting Information

# Thermo-mechanical Properties of Non-toxic Plasticizers for Polyvinyl Chloride Predicted from Molecular Dynamics Simulations

Snigdha S. Jagarlapudi#, Heaven S. Cross#, Tridip Das, William A. Goddard III*

Materials and Process Simulation Center (MSC), California Institute of Technology Pasadena, California 91125 USA

# These authors contributed equally

* To whom correspondence is to be addressed: wag@caltech.edu

Other Email addresses: snigdhaj@berkeley.edu; heavencross04@gmail.com; tridip@caltech.edu

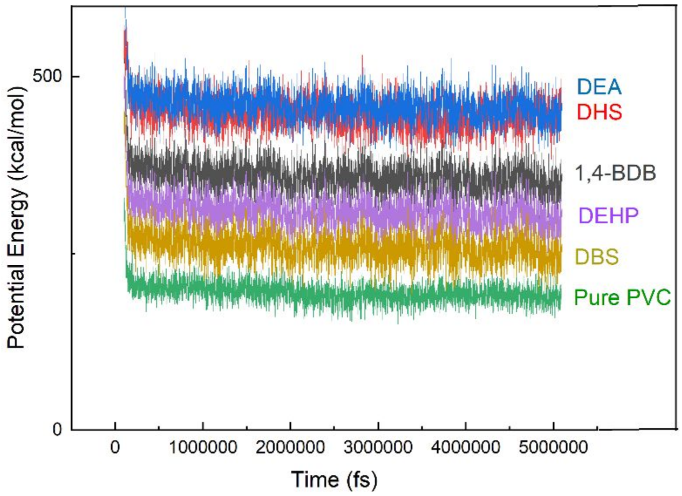


***Figure S1*** *Last five nanoseconds of the final 13 ns NPT heat-cooling cycle of equilibration, demonstrating convergence of the potential energy*

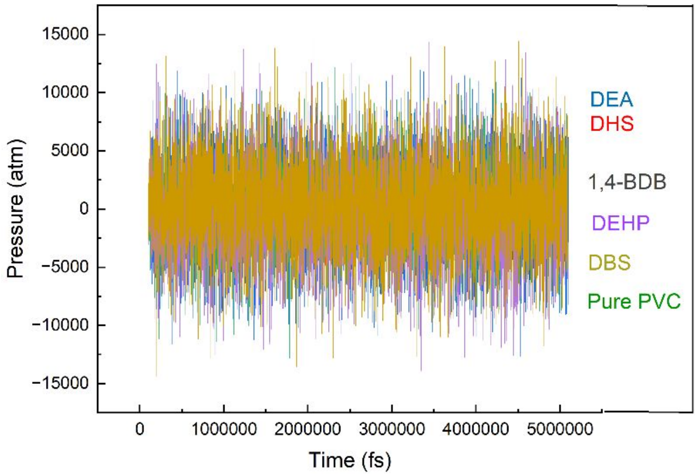


***Figure S2*** *Last five nanoseconds of the final 13 ns NPT heat-cooling cycle of equilibration, demonstrating convergence of the pressure, fluctuating around 0 atm.*

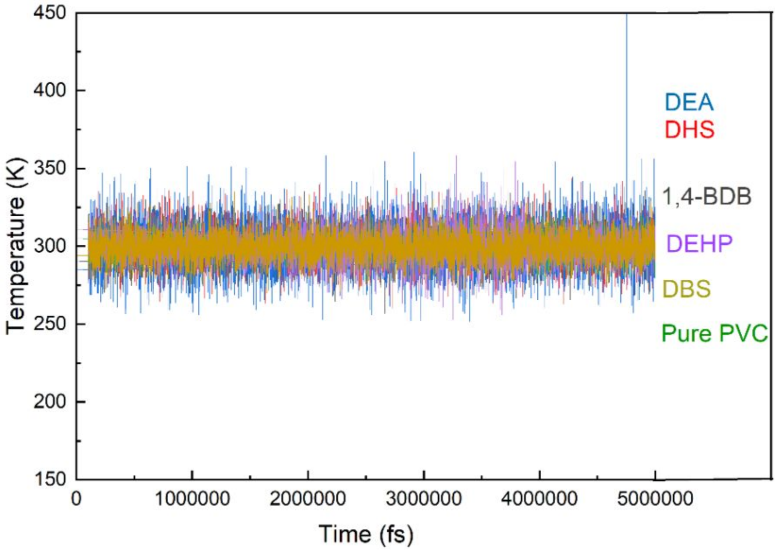


***Figure S3*** *Last five nanoseconds of the final 13 ns NPT heat-cooling cycle of equilibration, demonstrating convergence of temperature, fluctuating around 300 K*

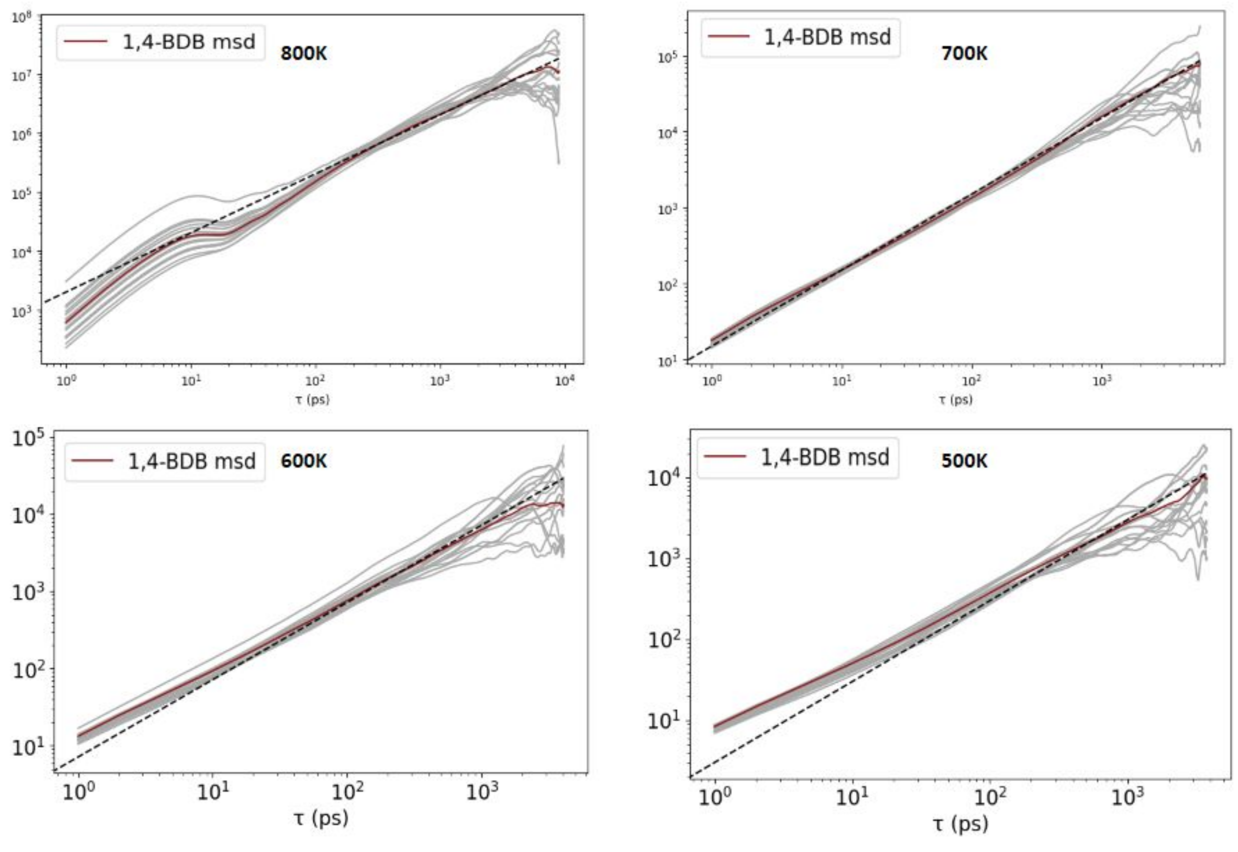


***Figure S4*** *The log(MSD) vs log(Time) plot to extract the diffusion constant for 1,4-BDB in PVC, NVT at 500K-800K. This system has 18 1,4-BDB molecules, the diffusion of which are all tracked individually and then averaged to get the diffusion coefficients (red BUTE MSD line). The dashed line has a slope of one. It is tangential to the MSD curve and its intercept gives 6D.*

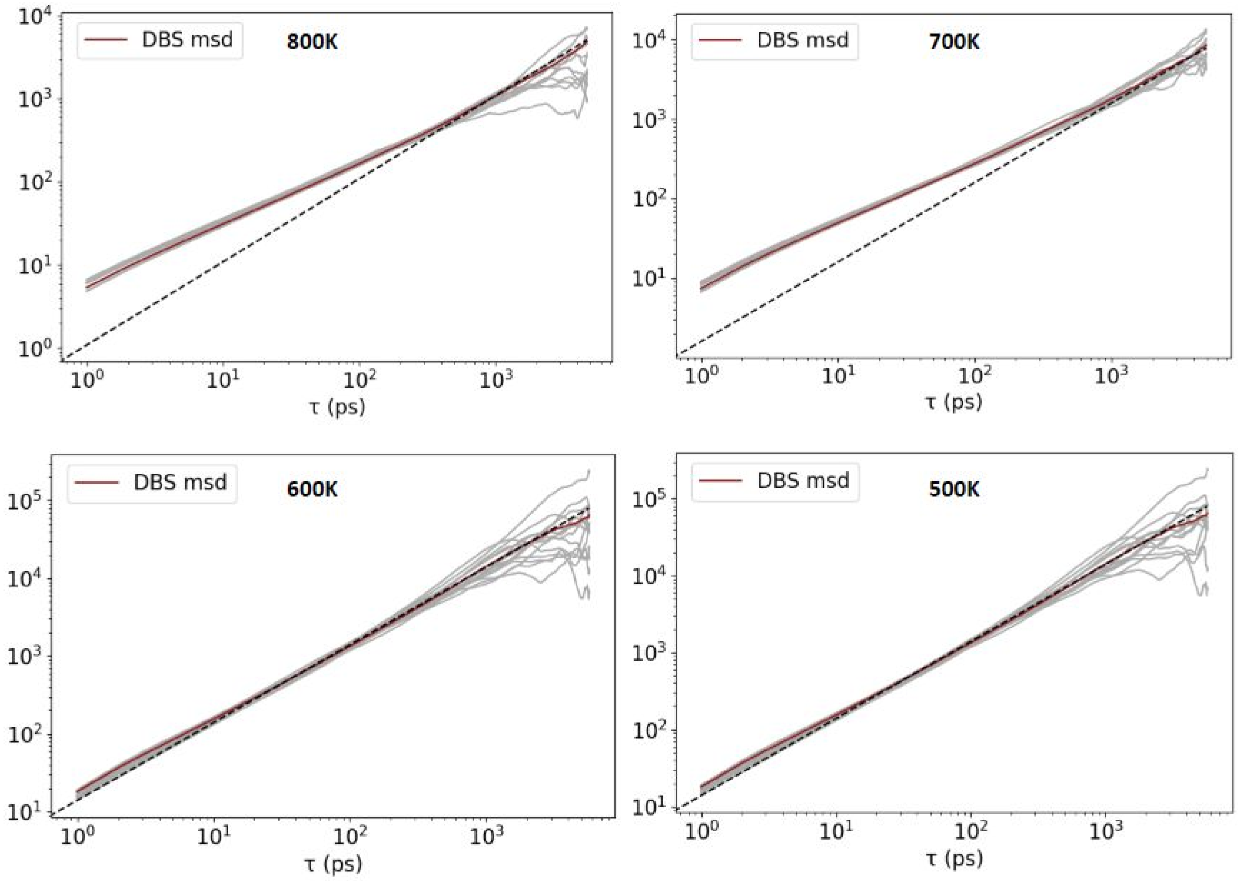


***Figure S5*** *The log(MSD) vs Log(Time) plot to extract the diffusion constant for DBS in PVC, NVT at 500K-800K. This system has 14 DBS molecules, the diffusion of which are all tracked individually and then averaged to get the diffusion coefficients (red DBS MSD line). The dashed line has a slope of one. It is tangential to the MSD curve and its intercept gives 6D.*

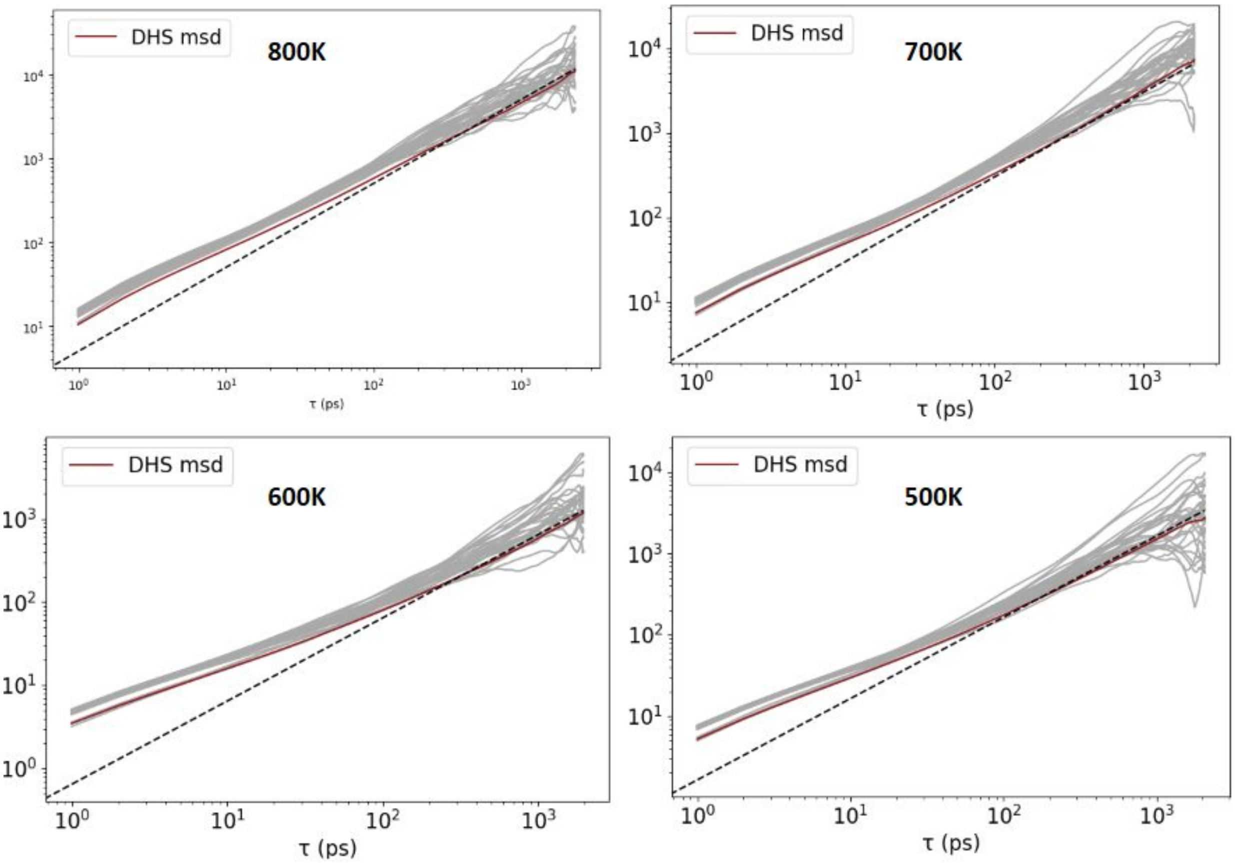


***Figure S6*** *The log(MSD) vs Log(Time) plot to extract the diffusion constant for DHS in PVC, NVT at 500K-800K. This system has 28 DHS molecules, the diffusion of which are all tracked individually and then averaged to get the diffusion coefficients (red DHS MSD line). The dashed line has a slope of one. It is tangential to the MSD curve and its intercept gives 6D.*

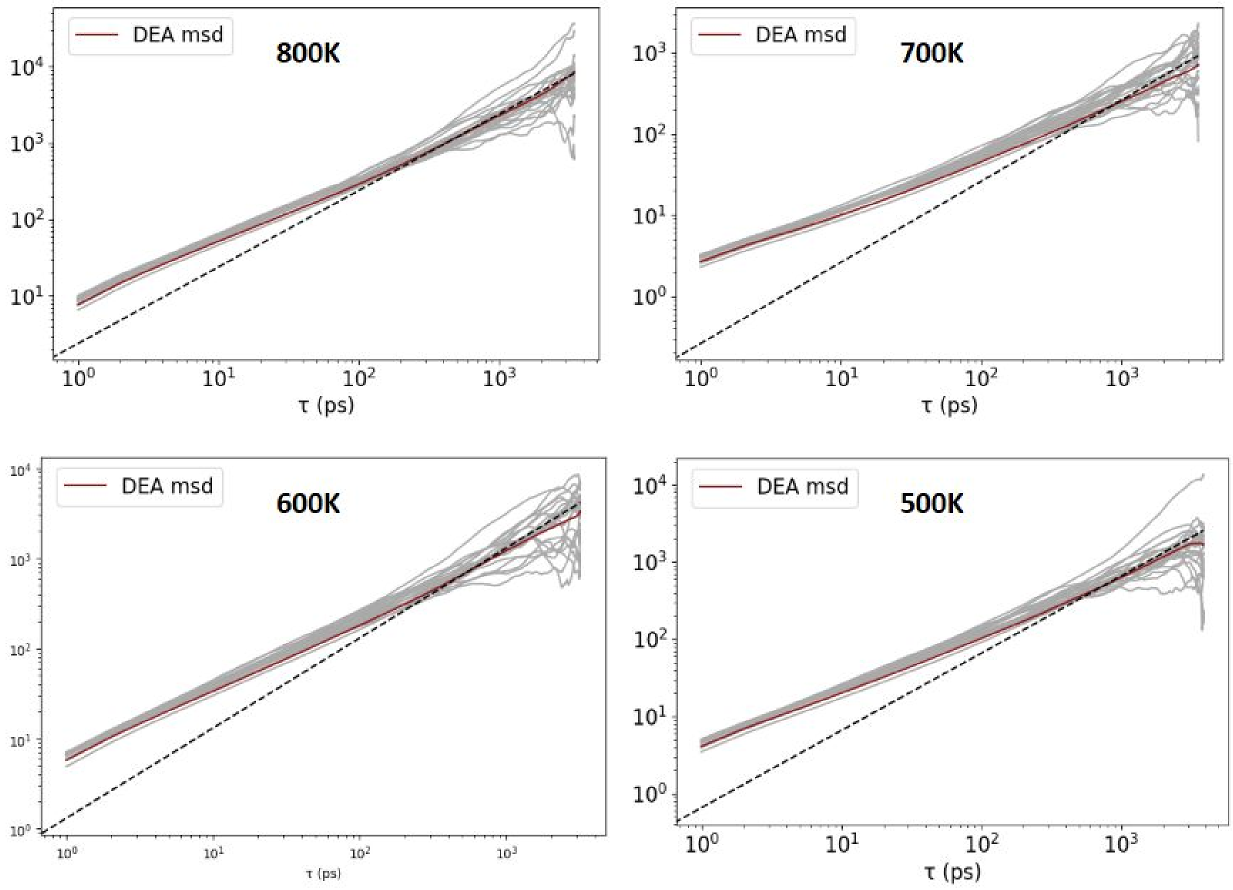


***Figure S7*** *The log(MSD) vs Log(Time) plot to extract the diffusion constant for DEA in PVC, NVT at 500K-800K. This system has 24 DEA molecules, the diffusion of which are all tracked individually and then averaged to get the diffusion coefficients (red DEA MSD line) for these 20nsNVT simulations. The dashed line has a slope of one. It is tangential to the MSD curve and its intercept gives 6D.*

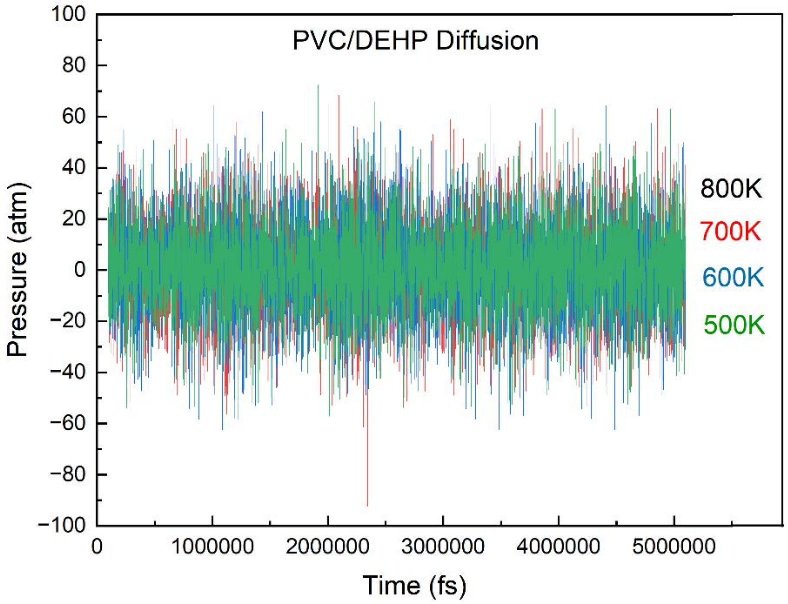


***Figure S8*** *Pressure fluctuations during the last five nanoseconds of the 20 ns PVC/DEHP NPT diffusion simulations*

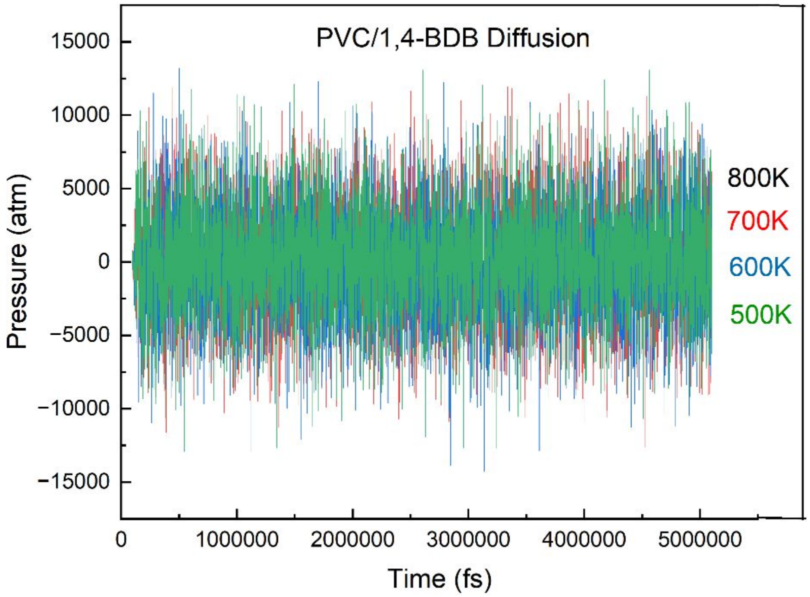


***Figure S9*** *Pressure fluctuations during the last five nanoseconds of the 20 ns PVC/1,4-BDB NPT diffusion simulations*

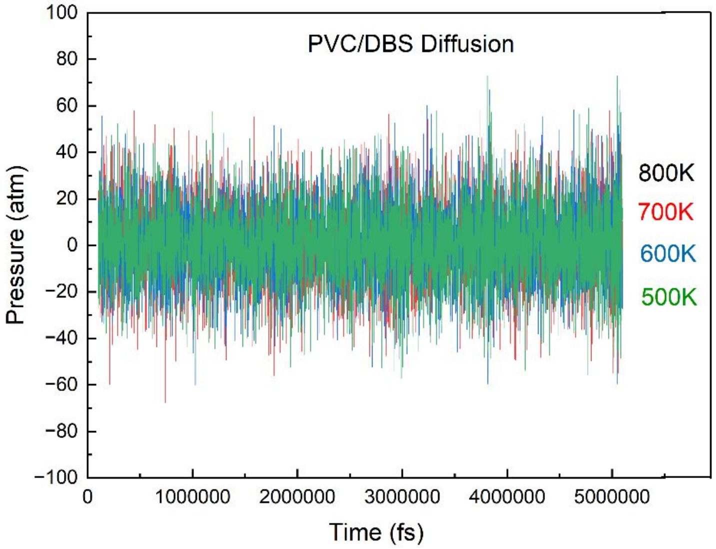


***Figure S10*** *Pressure fluctuations during the last five nanoseconds of the 20 ns PVC/DBS NPT diffusion simulations*

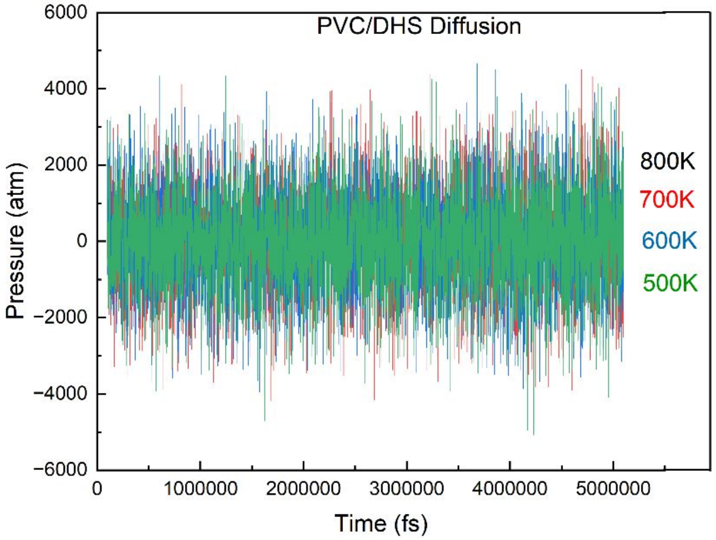


***Figure S11*** *Pressure fluctuations during the last five nanoseconds of the 20 ns PVC/DHS NPT diffusion simulations*

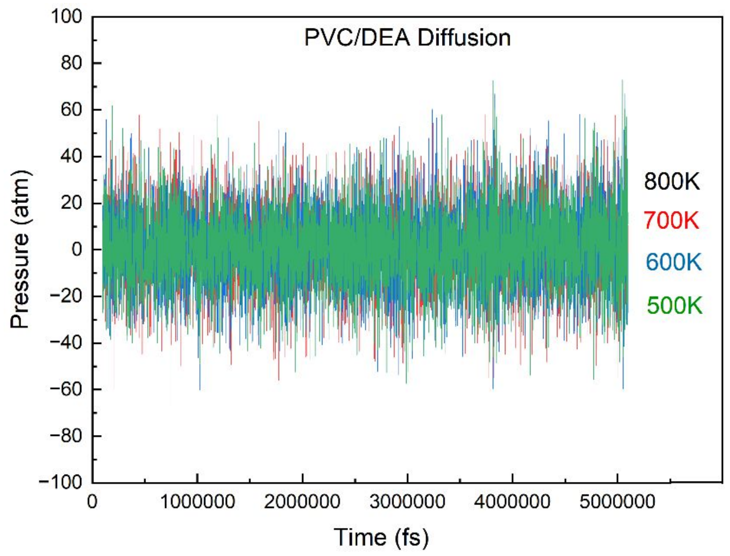


***Figure S12*** *Pressure fluctuations during the last five nanoseconds of the 20 ns PVC/DEA NPT diffusion simulations*

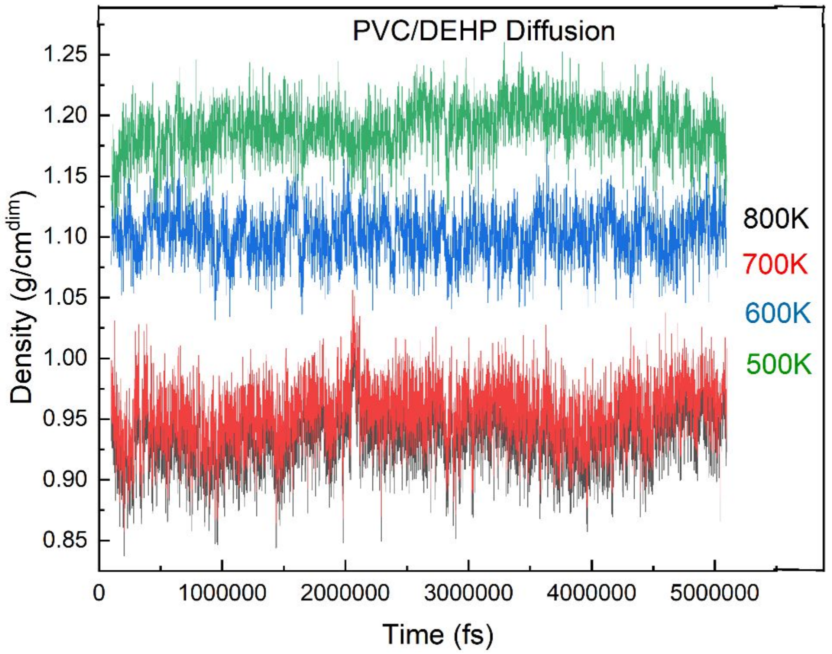


***Figure S13*** *Pressure fluctuations during the last five nanoseconds of the 20 ns PVC/DEHP NPT diffusion simulations*

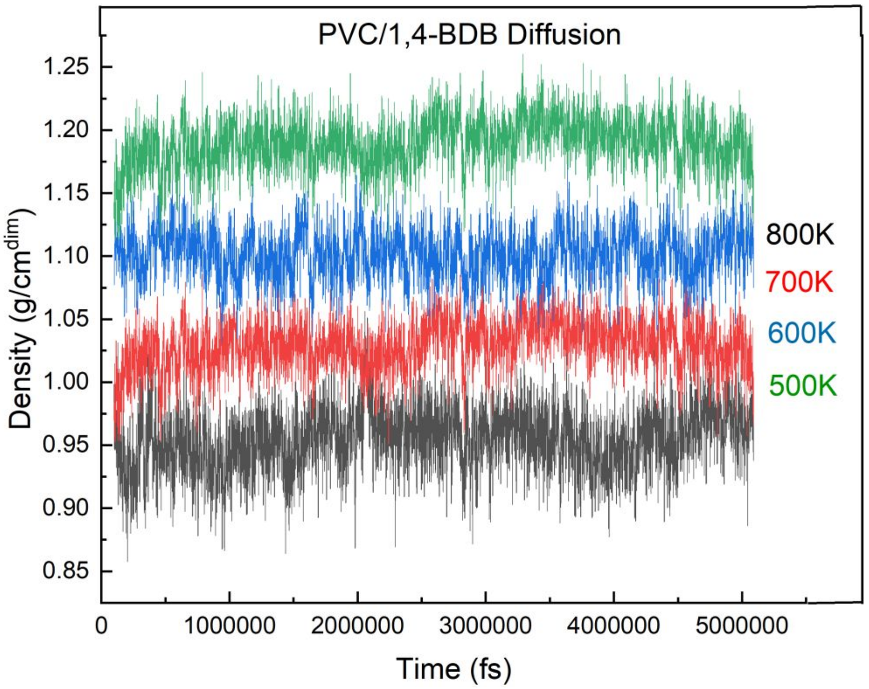


***Figure S14*** *Pressure fluctuations during the last five nanoseconds of the 20 ns PVC/1,4-BDB NPT diffusion simulation*

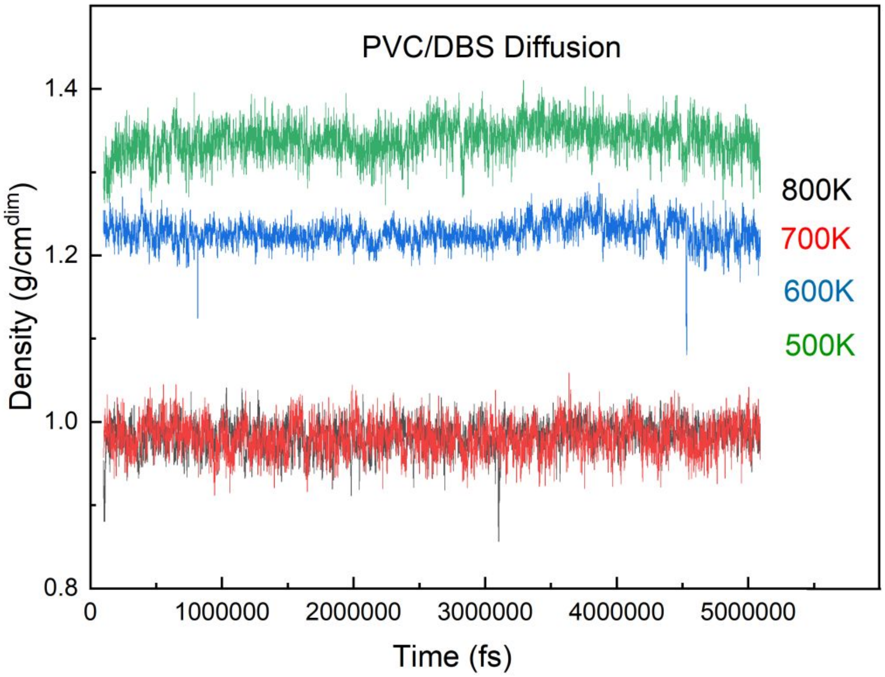


***Figure S15*** *Pressure fluctuations during the last five nanoseconds of the 20 ns PVC/DBS NPT diffusion simulations*

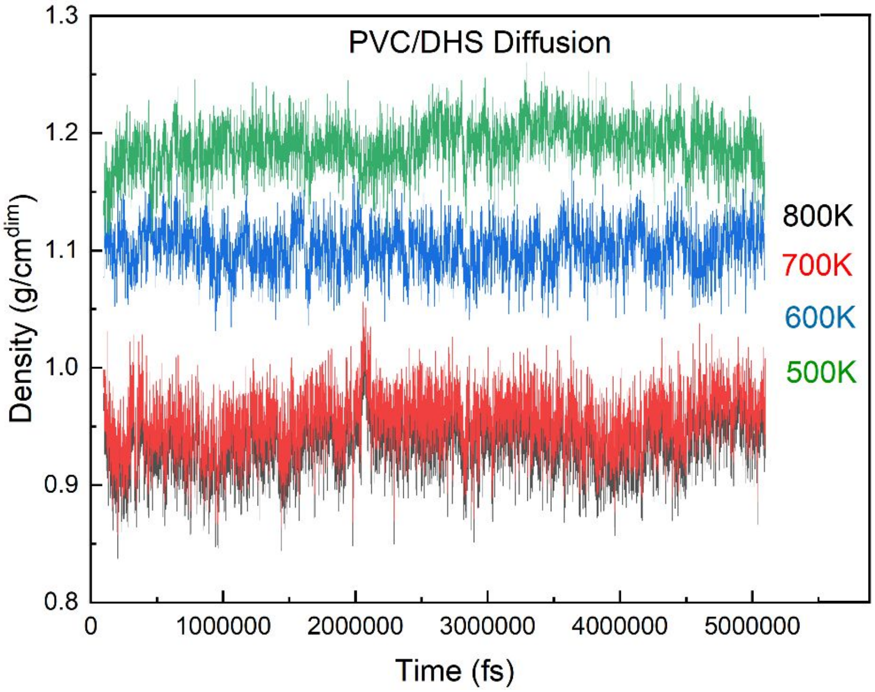


***Figure S16*** *Pressure fluctuations during the last five nanoseconds of the 20 ns PVC/DHS NPT diffusion simulations*

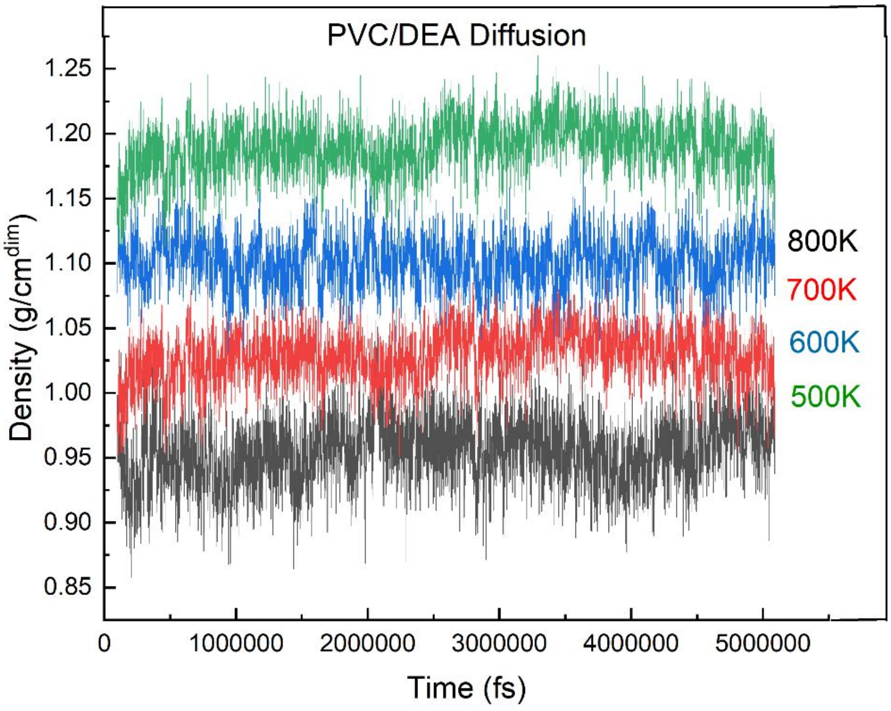


***Figure S17*** *Pressure fluctuations during the last five nanoseconds of the 20 ns PVC/DEA NPT diffusion simulations*

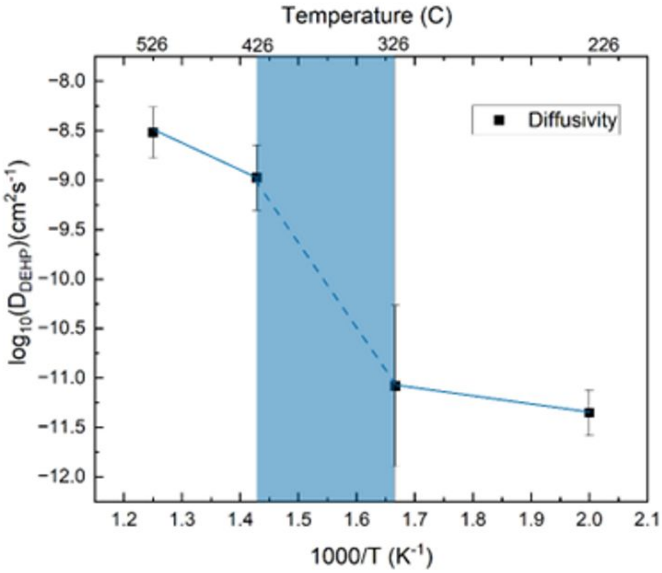


***Figure S18*** *Arrhenius plot of log(D) vs 1000/T for the diffusion coefficients of the DEHP-PVC system for temperatures 500K, 600K, 700K, and 800K. The graph demonstrates a strong, negative correlation with a distinct change in magnitude between 326 C and 426 C.*

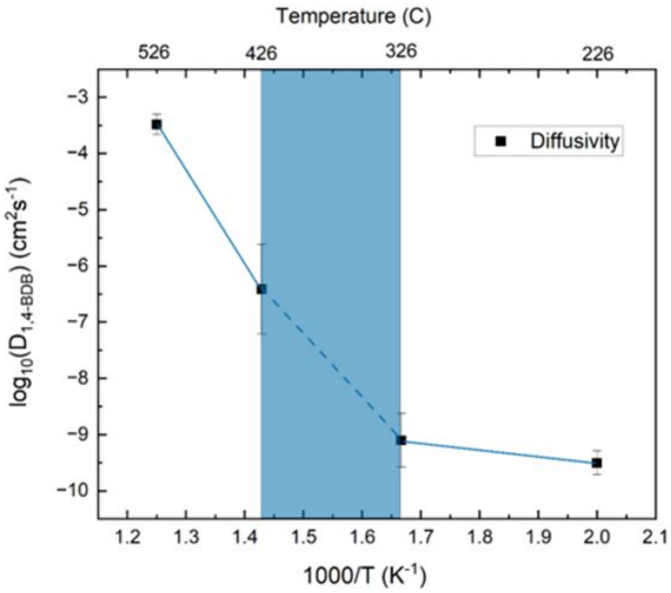


***Figure S19*** *Arrhenius plot of log(D) vs 1000/T for the diffusion coefficients of the PVC/1,4-BDB system for temperatures 500K, 600K, 700K, and 800K. The graph demonstrates a strong, negative correlation with a distinct change in magnitude between 326 C and 426 C.*

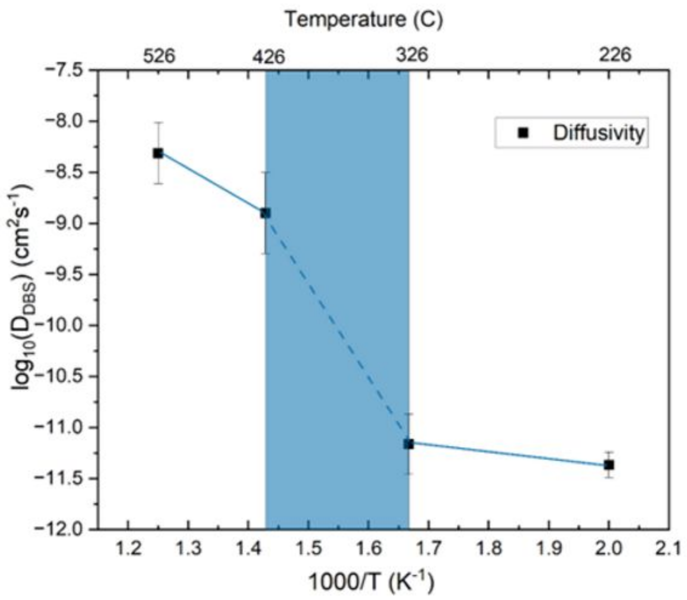


***Figure S20*** *Arrhenius plot of log(D) vs 1000/T for the diffusion coefficients of the PVC/DBS system for temperatures 500K, 600K, 700K, and 800K. The graph demonstrates a strong, negative correlation. The graph demonstrates a strong, negative correlation with a distinct change in magnitude between 326 C and 426 C.*

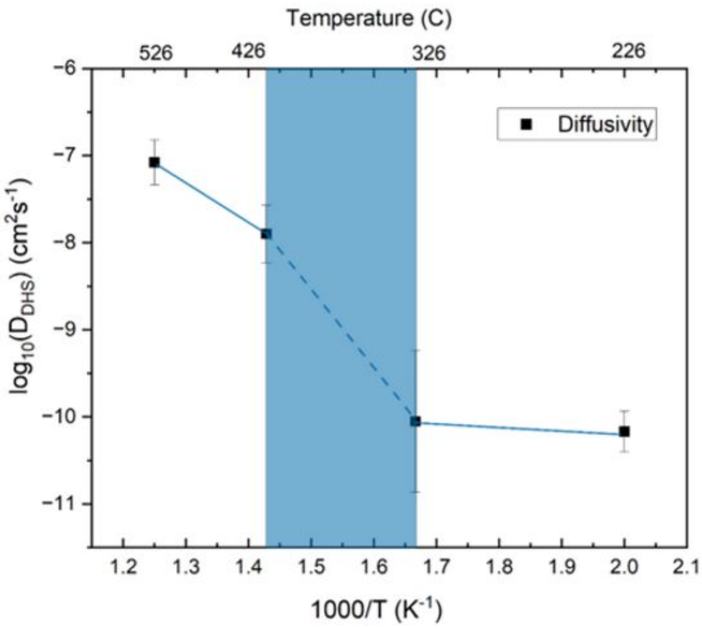


***Figure S21*** *Arrhenius plot of log(D) vs 1000/T for the diffusion coefficients of the PVC/DHS system for temperatures 500K, 600K, 700K, and 800K. The graph demonstrates a strong, negative correlation. The graph demonstrates a strong, negative correlation with a distinct change in magnitude between 326 C and 426 C.*

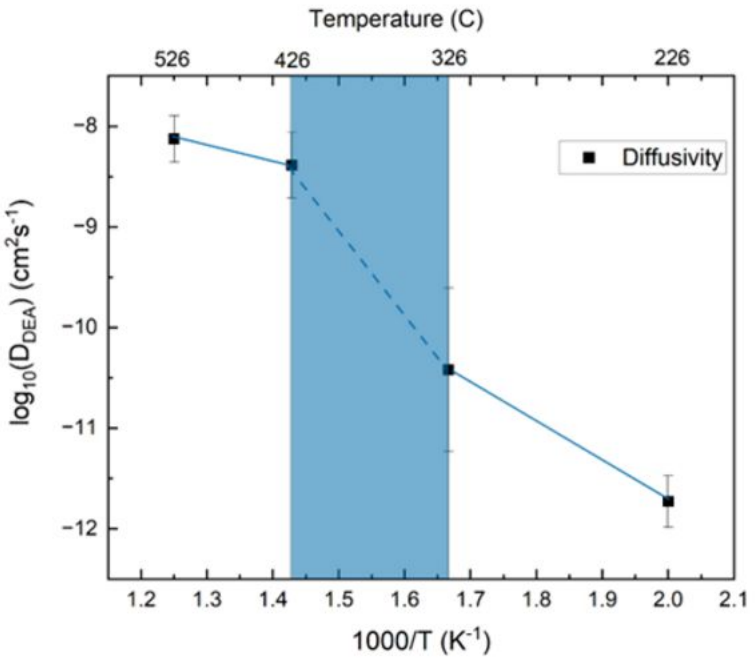


***Figure S22*** *Arrhenius plot of log(D) vs 1000/T for the diffusion coefficients of the PVC/DEA system for temperatures 500K, 600K, 700K, and 800K. The graph demonstrates a strong, negative correlation with a distinct change in magnitude between 326 C and 426 C.*
